# Examining Subtypes of Victimization in Saudi Arabia: A Comparative Analysis Across Gender Using PISA 2022

**DOI:** 10.3390/children13050589

**Published:** 2026-04-24

**Authors:** Georgios Sideridis, Mohammed H. Alghamdi

**Affiliations:** 1 Boston Children’s Hospital, Harvard Medical School, Boston, MA 02115, USA; 2 Department of Self-Development Skills, King Saud University, Riyadh 11362, Saudi Arabia; mhalghamdi@ksu.edu.sa

**Keywords:** bullying victimization, latent class analysis, person-centered methods, measurement invariance, gender differences, PISA, Saudi Arabia

## Abstract

**Background/Objectives:** Bullying victimization is a significant threat to adolescents’ psychological well-being and academic functioning. However, most prior research has relied on variable-centered approaches that may obscure meaningful heterogeneity in students’ victimization experiences. The present study aimed to identify latent subtypes of bullying victimization among adolescents in Saudi Arabia using nationally representative PISA 2022 data and to examine whether the structure and prevalence of these subtypes differed across gender. **Methods:** Data were drawn from the Saudi Arabian sample of the Programme for International Student Assessment (PISA) 2022 and included 6709 adolescents. Bullying victimization was assessed using 11 categorical indicators representing different forms of victimization. Weighted descriptive analyses were first conducted to estimate the prevalence of specific bullying behaviors. Multigroup latent class analysis (LCA) was then applied separately across gender to identify victimization profiles and evaluate measurement and structural invariance. Sequential invariance testing was used to determine whether the latent classes had equivalent meaning and prevalence across males and females. This study involved secondary analysis of an existing large-scale educational dataset and did not require trial registration. **Results:** Weighted descriptive estimates showed that the prevalence of specific bullying victimization experiences ranged from 7.5% to 24.3%, with boys reporting greater exposure than girls on most overt and coercive forms. Class enumeration supported a parsimonious three-class solution for both genders, reflecting low, moderate, and high victimization severity. Approximately 71–79% of students were classified in the low-risk group, 14–18% in the moderate-risk group, and 3–14% in the high-risk group. Measurement invariance testing supported full invariance of item-response probabilities across gender, indicating that the latent classes represented substantively comparable victimization patterns for males and females. In contrast, structural invariance was not supported, as males were more likely to belong to the high-victimization class, whereas females were more likely to be classified in the low-risk group. **Conclusions:** The findings indicate that gender differences in bullying victimization are attributable to differences in the level of exposure rather than differences in the underlying structure of victimization experiences. Bullying victimization appears to be concentrated within a relatively small but highly vulnerable subgroup of adolescents. These results support the importance of universal school-based anti-bullying policies and prevention initiatives, while also highlighting the need for targeted psychosocial support and protective interventions for students experiencing chronic or multiple forms of victimization.

## 1. Introduction

Bullying victimization during adolescence constitutes a persistent threat to students’ psychological well-being, academic engagement, and long-term developmental trajectories. Across countries and school systems, exposure to repeated peer aggression has been linked to elevated risks for depression, anxiety, psychosomatic complaints, school avoidance, and suicidal ideation, as well as diminished academic performance and school belonging [1,2,3]. These associations underscore that bullying is not merely an interpersonal or behavioral issue but a systemic barrier to equitable educational opportunity. Schools with high levels of peer victimization tend to have more negative school climates, lower academic functioning, and poorer overall student adjustment [4]. Thus, understanding the distribution of bullying among student populations—and identifying which students are at highest risk—is a key priority in educational policy.

The most widely accepted definition of bullying is a purposeful act of aggression that involves an inherent imbalance of power between the aggressor and victim, with the same ongoing perpetrator-victim dynamic occurring over time [3]. More recent conceptualizations oftentimes used in large-scale assessments understand bullying as a complex social phenomenon characterized by physical, verbal, relational, and increasingly digital forms of aggressive behaviors [5]. Within this framework, students may play the role of victims, perpetrators, and bully-victims—those who are both bullied as well as engage in bullying behavior themselves. Bully-victims represent a notably vulnerable group, with evidence suggesting they experience greater psychological distress and maladjustment compared to individuals engaged in either role only [6,7]. These distinctions highlight that bullying is not a monolithic experience but a heterogeneous phenomenon that needs nuanced conceptualization and measurement.

### 1.1. Global Data Systems

Much of the worldwide evidence base regarding bullying originates from large-scale cross-national monitoring systems. One of the most influential is the Health Behaviour in School-aged Children (HBSC) study, operating under the World Health Organization and including standardized measures of bullying victimization and perpetration since the 1993–1994 cycle [8,9]. HBSC uses a definitional framework based on that developed by Olweus and assesses bullying over a relatively recent (usually past couple of months) reference period, allowing for cross-national comparisons of prevalence and trends over time [5,9]. HBSC collects comparable data from nationally representative samples of adolescents across Europe, North America, and parts of Central Asia, and includes standardized questions on bullying victimization and perpetration based on the Olweus bullying questionnaire [9]. The core HBSC protocol typically assesses whether students have been bullied or have bullied others in the past two months, while additional optional modules capture specific forms of victimization such as relational, physical, or cyber bullying [8,10]. These long-running data collections have generated a substantial body of research examining cross-national patterns, gender differences, and developmental trends in peer victimization.

On the other hand, school bullying measures have only recently been added to the Programme for International Student Assessment (PISA) (since 2015), acknowledging the importance of school climate and student well-being as determinants of achievement [4]. In contrast to HBSC’s two-month reference period, PISA assesses students’ exposure to multiple forms of bullying over the preceding 12 months, including relational exclusion, verbal harassment, and physical aggression [4,11]. Although both systems are informative, they differ in several key methodological details, such as reference periods, response scaling, and operational definitions of bullying, a challenge well documented in the methodological literature [11,12].

### 1.2. Measurement Considerations

Importantly, there is significant global heterogeneity regarding how bullying is operationalized, which has direct implications for prevalence estimates and the classification of victimization profiles [12]. For instance, HBSC assesses bullying frequency with ordered response categories from “not at all” to “several times a week.” A common analytical practice in HBSC-based studies is to use a high-frequency threshold (e.g., “2–3 times a month or more”) to classify victims and perpetrators. This approach risks underestimating prevalence and misclassifying individuals with meaningful but less frequent exposure [13]. These operational decisions may produce skewed victimization profiles and biased associations with psychosocial outcomes. Other approaches—including those applied in PISA—preserve the full range of response categories, retain variation, and allow for more complex modeling of heterogeneity (e.g., creating a bullying intensity index) [11,13]. These discrepancies point to the relevance of thoughtful measurement frameworks when synthesizing cross-study results. They also emphasize the benefits of person-centered methods (e.g., Latent Class Analysis) that capitalize on the entire distribution of responses to model unobserved heterogeneity more effectively [14,15].

Empirical evidence supports that bullying in educational settings has become a serious problem in Saudi Arabia over the past few years, with levels of victimization comparatively high for an educational system [4]. In line with this, studies based on large-scale datasets like the Programme for International Student Assessment (PISA) and the Trends in Mathematics and Science Study (TIMSS) have reported that a significant percentage of these students indicate they have faced different types of aggression from their peers, including verbal, relational, or physical bullying [4,16]. Research in the Saudi context has focused on prevalence rates and associations with psychological well-being, academic performance, and school climate, all showing that victimization is associated with poorer adjustment, mental health problems, and supporting engagement [17,18]. However, this vast body of work has predominantly been restricted to variable-centered approaches that ignore the possibility of heterogeneous organization of victimization experiences [2] by treating bullying as a unidimensional construct.

Despite this policy relevance, much of the relevant literature relies on variable-centered approaches that treat bullying as a unitary latent construct. Although valuable and informative, these methods implicitly assume that bullying severity varies continuously and homogeneously across students, an assumption that may obscure meaningful heterogeneity and lead to information loss. Developmental and ecological perspectives, in contrast, suggest that victimization experiences cluster within subgroups of students who differ qualitatively in both the frequency and the form of exposure [5]. Empirical studies increasingly demonstrate that some students experience minimal exposure, others report occasional or situational victimization, and a smaller but consequential subset endures chronic or multi-form aggression [19,20].

Person-centered approaches, particularly latent class analysis (LCA), directly address this limitation by identifying discrete subpopulations characterized by shared response patterns rather than by average levels [14,15]. By explicitly modeling population heterogeneity, LCA has repeatedly shown that bullying involvement is better conceptualized as a set of distinct typologies rather than a single continuum. Across diverse cultural contexts, mixture models typically identify three to seven classes, including a large low-risk group and one or more small, highly victimized or “poly-victimized” subgroups [19,20]. Importantly, these high-risk categories, while numerically small, have a disproportionately high burden of psychological distress and absenteeism, and are at greater risk of academic failure [1]. Such findings indicate that prevention efforts may be more successful if they focus on specific, high-risk profiles rather than applying them uniformly to whole populations. Thus, identifying latent bullying typologies is not just a methodologically different approach but a necessary step toward understanding and treating problematic behaviors.

The issue is further complicated if gender is considered. Gender differences in bullying are well documented, but evidence indicates that these differences are not simply at the mean level. Boys are more often exposed to direct or physical forms of aggression, while girls are more often victims of relational or social exclusion behaviors [3]. Person-centered analyses suggest that these distinctions may manifest not only as variation in prevalence but also perhaps in the content or organization of victimization experiences [19,20]. Once class membership is established, the next step will be to determine whether latent classes are structurally equivalent across gender (i.e., whether they reflect the same outcome), a critical consideration for identifying whether interventions need to be broadly applicable or gender-specific.

These questions are of special relevance in the Saudi context of education, as Saudi schools are overwhelmingly organized along gender lines—boys and girls spend their formative years in separate institutions and peer environments—that may engender different social dynamics. In this respect, gender differences serve more as a comparison of two largely independent schooling ecologies than as (individual) differences within the same environment. This structure offers a distinctive opportunity to investigate whether bullying typologies are driven by universal developmental processes or are context-dependent, shaped by situational influences. From a policy standpoint, the question of whether profiles are invariant across gender (as opposed to distinct structures) directly informs how prevention resources will be allocated and designed.

### 1.3. Study Aims and Hypotheses

Despite increased attention to the importance of bullying as a problem in Saudi schools, there are significant gaps in understanding the varying nature and scope of victimization experiences within this context. In particular, it is unclear whether different subtypes of bullying involvement can be detected through exploratory person-centered methods and whether these typologies in terms of prevalence are similar for boys and girls within a gender-segregated school system. Filling these gaps will advance both theoretical understanding and targeted intervention efforts.

Guided by person-centered theory and prior mixture modeling research [19], the present study pursued three aims. First, we sought to identify latent subtypes of bullying victimization among Saudi adolescents using data from the Programme for International Student Assessment (PISA) 2022. Second, we evaluated whether the latent class structure was comparable across gender (configural similarity). Third, we formally tested measurement and structural invariance to determine whether gender differences reflected differences in class meaning or merely differences in prevalence. We hypothesized that (a) multiple discrete bullying profiles would emerge, characterized by low, moderate, and high victimization; (b) the same class structure would be observed across gender; and (c) class prevalences, but not measurement parameters, would differ between males and females.

## 2. Method

### 2.1. Participants and Data Source

Data were drawn from the Programme for International Student Assessment (PISA), an internationally standardized large-scale assessment administered triennially to nationally representative samples of 15-year-old students across participating education systems. PISA employs a two-stage stratified sampling design in which schools are sampled with probability proportional to size, and students are subsequently sampled within schools, yielding population-representative estimates when sampling weights are applied [11]. The present analyses included approximately N ≈ 5700 students with complete responses to the bullying victimization items and gender. Students self-identified their gender as male or female, which was used to define multigroup models. Consistent with PISA technical guidance, student weights were applied to all descriptive statistics and mixture estimations to ensure population-level inference.

The total sample included 3491 females and 3218 males. All models incorporated the final student sampling weight (W_FSTUWT) and accounted for school-level clustering to obtain nationally representative parameter estimates and appropriate standard errors. Inspection of item-level endorsement rates indicated substantial variability in reported bullying exposure, supporting the use of person-centered methods to identify latent heterogeneity in victimization experiences.

### 2.2. Administration Mode

The student questionnaire was administered under standardized conditions within participating schools, typically using computer-based assessment (CBA) platforms and under the control of local proctors. Although minor national adaptations may occur, data collection procedures are centrally coordinated by the OECD to ensure cross-national comparability and data quality.

### 2.3. Measure: Bullying Victimization Scale of PISA 2022

**Figure A1:**
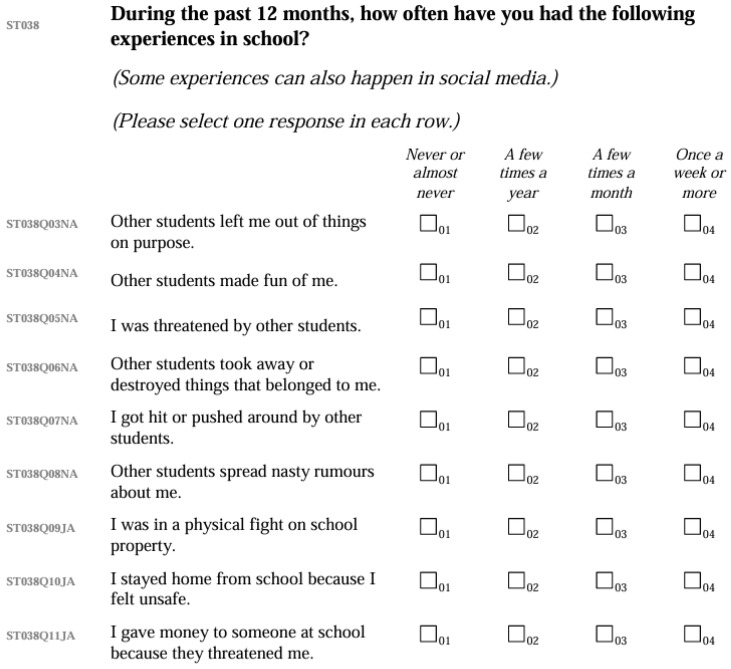
Bullying Scale Used in PISA 2022 Across Countries.

The bullying victimization scale in PISA 2022 (Figure A1) consists of a collection of items aimed at capturing exposure by students to different peer aggression experiences over the previous 12 months. The selected indicators encompass a range of bullying types, such as relational victimization (e.g., being excluded or a target of rumors), verbal victimization (e.g., being made fun of or threatened), physical victimization (e.g., being hit or pushed), and coercive/extortive behaviors (e.g., being forced to give money/belongings). This dimensional model is consistent with recent conceptualizations of bullying as encompassing a range of peer-aggression types that differ in visibility, severity, and function within the group context [2,3].

However, it should be noted that the PISA 2022 questionnaire does not provide an explicit definition of bullying (for instance, whether it implies a power imbalance or recurrence). Rather, bullying is operationalized by a list of behaviorally specific items measuring how often various types of victimization (e.g., exclusion, verbal harassment, physical aggression) have occurred in the past 12 months. This approach provides accounts of students’ lived experiences with peer victimization but may reflect a wider range of interpersonal aggression than definitions mathematically restricted to the Olweus framework. At the same time, the extended reference period may capture a broader range of victimization experiences, hence, providing a more comprehensive assessment of students’ exposure to peer aggression.

By including such items, the PISA framework enhances the ecological validity of coverage of students’ daily experiences in school contexts, encompassing both overt and covert victimization experiences that differentially impact students’ psychological and academic adjustment outcomes. The use of indicators across these domains allows the scale to identify multiple configurations of that victimization, rather than relying on a single aggregation measure.

Students responded to nine items assessing the frequency of peer victimization experiences (e.g., being made fun of, excluded, threatened, or physically hurt). Items were presented as a single matrix block with the stem: “During the past 12 months, how often have you had the following experiences in school?” The block also included the note, “Some experiences can also happen in social media.” Response options were as follows: Never; Almost never; A few times a year; A few times a month; and Once a week or more. The items captured multiple types of victimization experiences occurring at school, including exclusion, verbal harassment, threats, damage to belongings, physical aggression, rumor spreading, physical fighting on school property, school avoidance due to feeling unsafe, and extortion. Following standard latent class practice, interpretation focused on conditional response probabilities for endorsement of any bullying (Categories 2–4), computed as follows:

P(endorsement)=1−P(never)

This transformation facilitates the interpretation of class profiles on a common 0–1 probability metric.

### 2.4. Data Analyses

The main analytic approach selected was multigroup latent class analysis (LCA) because it aligned with the aims of identifying unobserved subpopulations and testing for gender comparability. In contrast with variable-centered approaches that estimate average associations across variables under the assumption of population homogeneity, LCA is an empirically driven tool for capturing heterogeneity in both responses and response patterns by estimating latent classes defined by particular covariation structures among observed behaviors [14,15].

The multigroup extension also formally tests for measurement and structural invariance, allowing one to assess whether identified profiles have the same meaning across gender and whether differences are due to profile structure or differences in class prevalence. This is especially relevant in Saudi Arabia, as gender-segregated schooling can lead to different peer surroundings between genders. Thus, multigroup LCA offers a sound and appropriate methodology to address both questions regarding the nature of bullying typologies and to assess potential gender differences in their structural and distributional properties.

Analyses proceeded in three sequential stages consistent with contemporary mixture modeling recommendations [14,15,19]:

1. Class enumeration within gender
2. Selection of a common, substantively defensible class structure
3. Multigroup latent class invariance testing

This staged approach avoids overextraction due to sample heterogeneity and ensures that subsequent invariance tests are conducted on a stable and interpretable latent structure.

#### 2.4.1. Latent Class Analysis Framework

Bullying typologies were estimated using multigroup latent class analysis (LCA), a finite mixture modeling approach that assumes population heterogeneity arises from an unobserved categorical latent variable C representing discrete subpopulations (classes). For a set of J categorical bullying indicators Y= ($Y_{1},\ldots ,Y_{J}$), the marginal probability of an observed response pattern is expressed as a weighted mixture of class-specific multinomial distributions:

$$P\left(Y_{i}\right)=\sum_{k=1}^{K}\;\pi_{k}\prod_{j=1}^{J}\;P\left(Y_{ij}|C_{i}=k^{k}\right),$$

where the following applies:

$\pi_{k}=P\left(C_{i}=k_{k}\right)$ denotes class prevalence and satisfies $\sum_{k=1}^{K}\;\pi_{k}=1$ , and $P\left(Y_{ij}|C_{i}=k_{k}\right)$ represents the conditional response probability for item j in class kt .

This formulation assumes local independence, such that indicators are independent within classes:

$$P\left(Y_{i}|C_{i}=k\right)=\prod_{j=1}^{J}\;P\left(Y_{ij}|C_{i}=k\right).$$

Model parameters, therefore, consist of the following:

1. Class prevalences $\pi_{k}$
2. Item-specific conditional probabilities $\theta_{\mathrm{jro}\;}=P\left(Y_{j}=r|C=c\right)$

Parameters were estimated by maximum likelihood via the Expectation–Maximization algorithm with robust standard errors.

#### 2.4.2. Multigroup Extension of LCA Model

To evaluate gender differences, the LCA was extended to a multigroup framework in which parameters were estimated separately for males (g=1) and females (g=2):

$$P\left(Y_{i}|G_{i}=g\right)=\sum_{k=1}^{K}\;\pi_{kg}\prod_{j=1}^{J}\;P\left(Y_{ij}|C_{i}=k,G_{i}=g\right).$$

This formulation allows both class proportions ($\pi_{kg}$) and item-response probabilities ($\theta_{\mathrm{jrog}\;}$).

The latent class enumeration process commenced with the estimation of separate LCAs for males and females to evaluate potential structural differences in latent heterogeneity. For each gender, models specifying one through six classes were estimated. Model selection considered multiple criteria: (a) Bayesian Information Criterion (BIC), (b) Sample-size adjusted BIC (SABIC), (c) Bootstrap likelihood ratio test (BLRT), (d) Minimum class size, and (e) Substantive interpretability of profiles. Given the large PISA sample size, statistical fit indices were interpreted cautiously, as information criteria tend to favor overextraction when sample sizes are large [21]. Consequently, parsimony and interpretability were emphasized. Solutions yielding extremely small classes (<5%) were scrutinized for stability unless profiles demonstrated clear theoretical coherence. Because mixture likelihood-ratio statistics tend to favor additional classes in large samples [21], model selection prioritized parsimony and stability over marginal improvements in information criteria. Although one female class was relatively small (~3%), inspection of conditional probabilities revealed a substantively coherent high-victimization profile that paralleled the corresponding male class. Because the class represented a meaningful subgroup rather than estimation noise, the three-class structure was retained for females as well.

#### 2.4.3. Multigroup Latent Class Invariance Testing

Measurement invariance across gender was evaluated through a nested sequence of models as described below.

*Model 1: Configural invariance*

A baseline model specified the same number of classes (K=3) across gender with all parameters freely estimated:

$$\pi_{kg},\theta_{\mathrm{jrog}\;}\;\mathrm{free}\;\mathrm{across}\;g.$$

This model tests whether a common qualitative class structure adequately represents both gender groups.

*Model 2: Measurement (threshold) invariance*

Item-response probabilities were constrained to be equal across gender:

$$\theta_{jrc1}=\theta_{jrc2}.$$

Under this model, classes have identical measurement properties and therefore equivalent substantive interpretation across groups. Differences between genders are attributed solely to class prevalence.

*Model 3: Structural invariance*

Class prevalences were additionally constrained:

$$\pi_{k1}=\pi_{k2}.$$

This model tests whether the distribution of students across classes is the same for males and females, respectively.

#### 2.4.4. Interpretation of Latent Profiles

Class profiles were interpreted using 0–1 endorsement probabilities and labeled descriptively based on severity:

1. Low or non-victimized
2. Moderate or occasional victimization
3. High or chronic victimization

Differences across gender were interpreted primarily as prevalence differences rather than differences in latent structure. All mixture models were estimated using Mplus (Version 9.3).

## 3. Results

### 3.1. Prevalence of Bullying Victimization

Weighted descriptive statistics were explored to depict the prevalence and severity of victimization in the total Saudi PISA 2022 sample prior to estimating latent classes. The most common experience reported was being made fun of by 24.3% of students at least a few times in the last 12 months. This was followed by spreading nasty rumors about them (16.9%), being deliberately excluded from things (13.1%), and preferring to stay home rather than go to school because they feel it is not safe for them there (11.3%). Rates of being in a physical fight on school property (9.9%), having possession taken away or destroyed (9.4%), being threatened (9.3%), being hit or pushed around (8.2%), and giving money to someone because of threats (7.5%) were lower but still significant.

The prevalence of monthly-or-more victimization was 3.1% for physical fights and 8.2% for being made fun of. Weekly-or-more exposure was rarer overall but was still nontrivial for some indicators, especially giving money due to threats (4.0%) and being made fun of (3.2%).

Several types of victimization showed marked gender differences. For most indicators, boys were more likely than girls to report experiencing them, particularly the more overt and coercive experiences. Boys were significantly more likely than girls to say they had been made fun of (28.8 percent vs. 20.4), threatened (13.8 vs. 5.3), had belongings taken or destroyed (12.7 vs. 6.6), hit or pushed (12.3 vs. 4.5) involved in a physical fight on school property (16.2 vs. 4.4) and gave money because someone threatened them (12.1 vs. 3.4). For social exclusion (13.3% boys, 13.0% girls), rumor spreading (17.3% vs. 16.5%), and staying home because of feeling unsafe (11.5% vs. 11.2%) there were few gender differences.

Weighted item-level prevalence estimates also showed which types of victimization were most prevalent and where gender differences were greatest (see Table 1). The top three experiences were being made fun of (24.3%), nasty rumors being spread about them (16.9%), and being excluded on purpose (13.1%). In contrast, the most overtly aggressive and coercively painful forms of victimization exhibited the largest gender differences. Boys reported significantly higher rates than girls for being involved in a physical fight on school property (16.2 percent vs. 4.4 percent), giving money due to threats (12.1 percent vs. 3.4 percent), being threatened (13.8% vs. 5.3%), being hit or pushed around (12.3% vs. 4.5%) and having belongings taken away or destroyed (12.7% vs. 6.6%). In comparison, the gender differences observed for exclusion, rumor spreading, and staying home because of feeling unsafe were comparatively smaller (although statistically significant in the weighted analyses). Thus, this descriptive pattern supports the idea that boys were disproportionately exposed to more direct, physical, and coercive forms of victimization; on the other hand, some relational and school-avoidance indicators displayed much smaller gender gaps.

### 3.2. Latent Class Enumeration Process

Latent class models delineating one to eight classes were fit separately for females and males. Log-likelihood (LL), Bayesian Information Criterion (BIC), sample-size-adjusted BIC (SABIC), entropy, bootstrap likelihood ratio tests, minimum class size, and substantive interpretability guided model selection. Since information criteria are prone to over-extraction of classes in large samples, greater emphasis was placed on parsimony, stability, and meaningful profile differentiation rather than on incremental increases in fit statistics.

### 3.3. Latent Class Solution in Females

In females (*n* = 3491), information criteria decreased as classes were added; however, improvements diminished after three classes, and additional solutions resulted in very small subgroups that were likely artifacts of enhanced power and had little interpretable validity. The three-class model yielded SABIC = 2116.34 and entropy = 0.853, indicating adequate fit and classification precision. Class proportions were approximately 79.1%, 17.7%, and 3.1% of the total sample. Although the smallest class fell slightly below the conventional 5% heuristic, inspection of conditional response probabilities revealed a coherent, distinct profile characterized by high endorsement across all bullying indicators, which is well aligned with a low-prevalence class. In contrast, models with four or more classes produced multiple classes below 5% that likely reflected unstable subgroups confounded by measurement error, suggesting statistical overextraction rather than additional meaningful typologies. Accordingly, the three-class solution was retained for females and reflected the most optimal pattern for this group (see Table 2).

### 3.4. Latent Class Solution in Males

For males (*n* = 3218), the three-class solution provided the most parsimonious and interpretable representation of the data. Entropy was 0.860, indicating good classification quality, and class proportions were well-balanced (71.2%, 14.9%, 13.9%), with all classes exceeding recommended minimum size thresholds. Although information criteria continued to decline slightly for higher-class solutions, these models produced small or redundant classes (<5%) with limited substantive differentiation and utility. Therefore, consistent with parsimony and interpretability criteria, the three-class solution was selected for males (see Table 3).

As both genders yielded interpretable three-class solutions with comparable profile shapes, a common three-class structure (K = 3) was adopted for multigroup analyses. Importantly, the small female high-victimization class closely mirrored the corresponding male high-risk profile in its conditional probability pattern, indicating that it represented a low-frequency but meaningful subgroup rather than a spurious statistical artifact. Adopting a common class structure enabled formal tests of measurement invariance and direct comparisons of class prevalence across gender.

### 3.5. Interpretation of Latent Class Profiles

To facilitate interpretation, conditional response probabilities were transformed to 0–1 endorsement probabilities representing the likelihood of experiencing any bullying (i.e., 1−the probability of “never”). Across both genders, class profiles followed a clear monotonic severity gradient.

**Figure 1:**
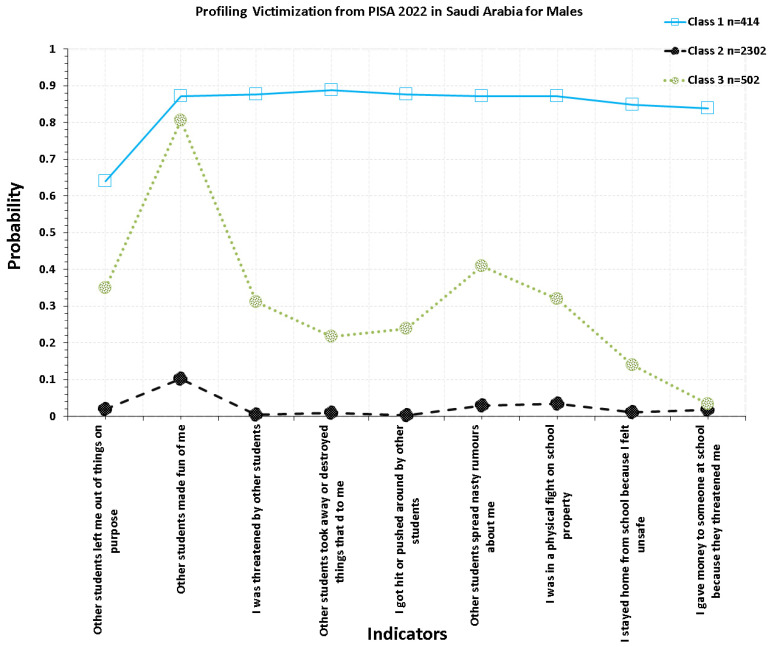
Optimal Latent Class Solution for Males in Saudi Arabia Using PISA 2022.

**Figure 2:**
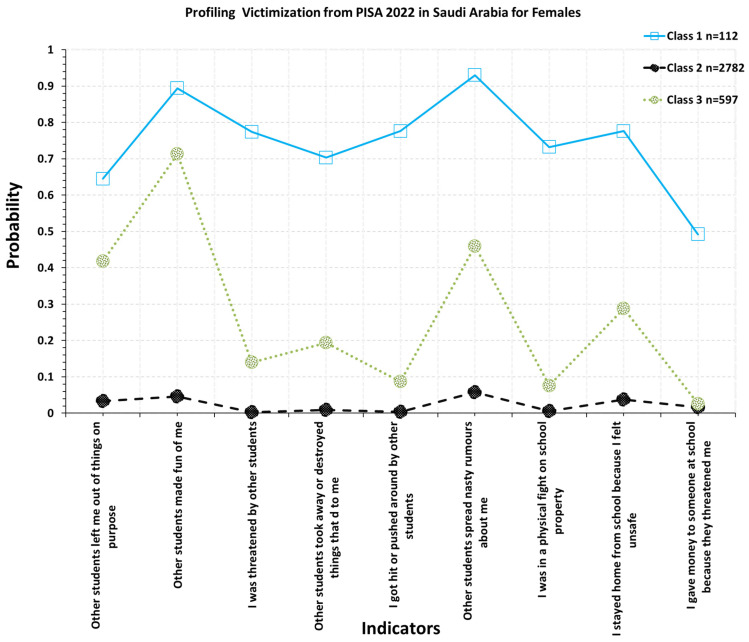
Optimal Latent Class Solution for Females in Saudi Arabia Using PISA 2022.

Upon closer inspection of the class-specific endorsement probabilities, the upper two classes differed not only in severity but also in substantive patterning (see Figure 1 and Figure 2). In both genders, Class 1 reflected a poly-victimization profile, with elevated probabilities across almost all bullying indicators. Among girls, this class was characterized by especially high endorsement of having belongings taken away or destroyed, spreading of nasty rumors, being hit or pushed, involvement in physical fights, exclusion, and being threatened. In boys, however, the same class displayed a pattern with high endorsement of staying home because of feeling unsafe, exclusion, physical fights, property-related victimization, threats, and giving money because of threats. Thus, in boys, the high-victimization class represented a chronic and multi-form exposure.

In contrast, Class 2 showed a more selective, qualitatively narrower profile. In girls, this subgroup was characterized mostly by rumor spreading, being made fun of, and property-related victimization. In boys, the respective moderate-victimization class was also driven by rumor spreading and being made fun of. However, it additionally showed moderate probabilities of exclusion, threats, and physical fights, suggesting a somewhat broader configuration than in girls. These findings substantiate the conclusion that the two upper classes were not different in severity but also in the configuration of bullying experiences.

**Class 1: High victimization class**

Students in this class displayed elevated endorsement probabilities across nearly all bullying indicators, consistent with multi-form victimization. Although this class was less prevalent, it represented the subgroup with the highest overall risk.

**Class 2: Low victimization class**

Students in this class demonstrated near-zero endorsement probabilities across the bullying indicators, suggesting minimal or no exposure to victimization.

**Class 3: Moderate victimization class**

Students in this class showed selective elevation on several bullying indicators, indicating occasional or situational victimization rather than pervasive exposure across all forms.

### 3.6. Gender Differences in Class Membership

Despite structural similarity, class prevalence differed across gender. Males were disproportionately represented in the high-victimization class (≈14%), whereas females were more frequently classified in the low-risk class. These differences indicate that gender disparities primarily reflect differences in the distribution of students across risk strata rather than differences in the underlying structure or meaning of the latent classes.

### 3.7. Measurement Invariance Across Gender

Measurement invariance of the three-class bullying typology across gender was evaluated using a sequential multigroup latent class modeling framework. Following established mixture-modeling practice, we estimated (a) a freely estimated configural model in which item-response probabilities and class prevalences were allowed to vary across gender, (b) a semi-constrained model in which the latent class measurement parameters (conditional response probabilities) were constrained equal across groups while class sizes remained free, and (c) a fully constrained model in which both measurement parameters and class proportions were constrained to equality [14,19].

#### 3.7.1. Configural Invariance (Freely Estimated Model)

The baseline model demonstrated good convergence and acceptable classification precision. Fit indices were AIC = 51,908.66, BIC = 53,046.14, and sample-size-adjusted BIC = 52,515.45, with an entropy for the bullying latent class variable of 0.853, indicating adequate separation among classes. The overall class distribution across the combined sample was 8.1% (Class 1), 18.1% (Class 2), and 73.8% (Class 3). Gender-specific transition probabilities suggested modest differences in prevalence, with females showing probabilities of 0.035/0.196/0.770 across Classes 1–3 and males 0.132/0.166/0.703. These results indicate that the same three latent profiles were identifiable in both groups, supporting configural equivalence of the class structure.

#### 3.7.2. Measurement Invariance (Semi-Constrained Model)

Constraining conditional item-response probabilities to equality across gender produced identical log-likelihood and information criteria to the freely estimated model (ΔLL = 0; AIC/BIC unchanged), indicating no deterioration in fit. Entropy remained 0.853, and class proportions were virtually unchanged. Because equality constraints on the measurement parameters did not worsen model fit, the bullying indicators appear to function equivalently across males and females. This finding supports measurement (profile) invariance, suggesting that the latent classes have the same substantive meaning in both genders.

#### 3.7.3. Distributional Invariance (Fully Constrained Model)

A more restrictive model additionally constraining latent class prevalences resulted in a decrease in fit, with LL = −26,158.58 compared to −25,787.33 for the semi-constrained model (ΔLL ≈ 371), and higher AIC (52,485.16) and BIC (53,057.30) values. Entropy declined slightly to 0.846. These changes indicate that forcing equal class sizes across gender meaningfully degrades model fit. Thus, while measurement invariance is supported, distributional invariance is not, implying that males and females differ in the prevalence—but not the structure—of bullying profiles.

Overall, the invariance results demonstrated that (a) the same three latent bullying subtypes are present for both genders and (b) the item-response patterns defining each class are statistically equivalent, enabling substantively valid comparisons. However, the proportion of students assigned to each class varied by gender, suggesting differential exposure or vulnerability rather than differences in measurement.

## 4. Discussion

The present study applied multigroup latent class analysis to nationally representative data from Saudi Arabian students participating in Programme for International Student Assessment (PISA) 2022 to identify distinct bullying victimization typologies and to evaluate their comparability across gender. Several findings emerge with both theoretical and practical significance. First, bullying involvement was not distributed continuously across students but instead organized into three qualitatively distinct subgroups characterized by low, moderate, and high levels of exposure. Second, these latent profiles demonstrated measurement invariance across gender, indicating that the same behavioral patterns define classes for males and females. Third, despite structural similarity, class prevalences differed substantially, with males disproportionately represented in the high-victimization subgroup. Collectively, these findings suggest that gender differences in bullying reflect differences in the distribution of risk rather than differences in victimization experiences themselves.

### 4.1. Latent Heterogeneity in Bullying Exposure

Although the pattern of three latent profiles appears consistent with a severity-based gradient of bullying exposure, close inspection reveals that these classes also diverge qualitatively by configuration and co-occurrence of victimization experiences. Therefore, while endorsement probability increases monotonically across classes, the high-victimization subgroup is defined not just by frequency but by a poly-victimization pattern in which students are simultaneously engaging in relational, verbal, and physical forms of aggression. This finding aligns with prior research suggesting substantial overlap among bullying modalities and supports the conceptualization of victimization as a generalized risk experience rather than a set of discrete subtypes [5,22]. These students are also known to face considerable psychosocial and academic consequences, including internalizing symptoms, disengagement from school, and low achievement [1,2]. This pattern is theoretically significant given research suggesting that facing cumulative exposure across different domains has been associated with disproportionately increased psychosocial risk compared to experiencing any one context of victimization in isolation [1].

The moderate class, on the other hand, seems to reflect a situational or context-dependent victimization pattern, with selective endorsement of certain behaviors rather than universal experience across all indicators. This pattern suggests that those students might be embedded in peer environments where bullying is episodic or contingent upon particular social dynamics, rather than being chronic and endemic. Thus, the latent class structure should not be interpreted as a sequential progression of severity but can rather be understood as categorizing qualitatively different profiles in the organization of victimization across time and space—identity categories representing either limited exposure (some), situational involvement (some), or cumulative multi-form victimization (more). This separation is consistent with person-centered theory, which holds that subgroups differ in the extent and structural patterning of experiences [14,15]. Thus, the added value of the person-centered approach lies not simply in identifying severity strata but in revealing distinct configurations of risk that may require different explanatory frameworks and intervention strategies.

### 4.2. Gender Similarities and Differences

These results are generally consistent with previous person-centered research on bullying, which often has found a three-class solution of low-risk, moderate- and high-or poly-victimized groups across populations [19,20,23]. As observed in these studies, the high-risk subgroup tends to encompass a smaller proportion of the general population while experiencing increased rates of co-occurring forms of victimization (e.g., physical, verbal, and relational bullying) [2,20]. Supportive of the robustness of the three-class typology, the convergence of findings across diverse samples and educational contexts indicates that core patterns of bullying involvement may generalize to other school systems and cultural settings [19,23].

Furthermore, by showing that these bullying victimization profiles replicate and exhibit measurement invariance across gender contexts (gender-segregated schooling), the present study contributes to the emerging literature supporting cross-group comparability of latent bullying typologies. It indicates a broader realm in which person-centered approaches can be applied in the study of bullying experiences [19,24].

The reported gender differences in class prevalence should be seen in the wider sociocultural and institutional context of Saudi education, which is mostly gender-segregated. In this context, boys and girls are embedded in mostly separate peer ecologies featuring different social norms, interactional dynamics, and behavioral expectations. This structural separation has the potential to form context-specific peer cultures that influence the frequency and nature of bullying behaviors [25].

Boys’ school environments may allow peer interactions to be more strongly organized around dominance hierarchies and norms of masculinity that tolerate or even condone overt forms of aggression, including physical and verbal confrontation [2,3]. These dynamics might prime repeated and multi-form victimization, which in turn offers a better explanation for the higher prevalence of the high-risk class among males. On the contrary, girls’ peer space might value more relational ties and indirect social control, which lowers the incidence of victimization but still allows, e.g., exclusion from the group and gossip.

The item-level descriptive findings and class-specific response patterns converged in showing that boys were more exposed to direct, physical, and coercive forms of victimization. In contrast, relational and verbal forms were common in both genders and were especially central to the moderate-risk profiles. This pattern helps explain why males were disproportionately represented in the high-victimization subgroup, even though the overall latent structure remained comparable across gender. It is noteworthy that the current results demonstrate that these differences in the context of exposure do not alter the structure, at least at the aggregate level, of victimization profiles associated with gender. This finding might indicate that the common structural nature of bullying experiences reflects generalizable developmental processes. Meanwhile, discrepancies in prevalence are more likely to be ascribed to locally rooted peer norms and institutional arrangements. In conclusion, the gender differences identified in this study should not be seen as individual—level properties but instead reflect different social ecologies of gender—segregated school systems.

### 4.3. Implications for Educational Policy

These findings have significant policy implications. In relation to international assessments such as the Trends in International Mathematics and Science Study, Saudi Arabia has been shown to have relatively high bullying rates, thus being classified as a high-prevalence system internationally. In these contexts, universal anti-bullying programs may indeed be needed. Results from the person-centered approach suggest that risk of victimization is not evenly distributed across all students, but rather reflects substantive subgroups [19,20]. Therefore, educational policy must evolve beyond universal (population-wide) strategies to a multi-tiered system that combines broad prevention with intensive support for students at risk. A key element of such an approach is the systematic identification of students exposed to chronic or multi-form victimization. In practice, this might be operationalized via short, standardized screening tools administered periodically at the classroom- or school-level, building from validated items similar to those used in large-scale assessments such as PISA. Such tools can offer efficient, low-burden methods for identifying broader patterns of victimization across multiple domains. Also, assessments by teachers and structured observation of peer interaction can be additional sources of information, especially for children who are likely to underreport victimization in self-reports. It may improve identification efforts, for example, by combining data from student self-reports with teacher observations and administrative records (e.g., absenteeism). Within the MTSS approach, students were flagged as falling within moderate or high-risk profiles. They could, relative to their peers in a given cohort, receive evidence-based interventions, such as social-emotional skills training, counseling, or specific peer-to-peer support programs. At the same time, universal prevention strategies continued at the school level.

### 4.4. Practical Implications for Teachers and Schools

At the classroom level, the current findings emphasize the clear need for structured, ongoing monitoring of peer interactions. Short, periodic check-ins or survey-based assessments can be used by teachers to identify students who are experiencing different forms of victimization (i.e., exposure to multiple forms within the same timeframe). Additionally, continuing professional development (CPD) programs that train teachers to become aware of behavioral and social indicators of child victimization—examples might include withdrawal, changes in preference for peer groups that have a proclivity towards avoidance behaviors—may be beneficial. Crucially, identification efforts should not depend entirely on chance revelation; rather, they should be integrated into standardized, systematized screening practices that facilitate proactive intervention.

### 4.5. Methodological Strengths

The current study has several methodological strengths. Nationally representative PISA data add to generalizability across the Saudi student population. A large sample size allows stable estimation of the mixture profiles and accurate parameter recovery. Inference at the population level is preserved through the application of sampling weights and clustering by classroom. Finally, formal multigroup invariance testing provides stronger evidence of cross-gender comparability than descriptive or separate analyses alone.

### 4.6. Limitations and Future Directions

Despite these strengths, several limitations warrant consideration. First, bullying indicators relied on student self-reports, which may be subject to recall bias or social desirability effects. Second, the cross-sectional nature of this design prevents making conclusions about the temporal stability and developmental evolution of the identified latent profiles. The classes identified in the present study should therefore be viewed as characteristics of victimization at a given point in time, rather than as persistent subgroups. It is still not known whether students who are classified as moderate- or high-risk profiles remain in those categories over time or shift between levels of exposure based on changing peer environments or developmental processes. Third, the 12-month reference period in PISA does not provide information on school mobility. Hence, it is impossible to determine whether experiences of victimization reflect the current school or a prior educational setting. Fourth, although the smallest female class was substantively interpretable, its modest size may reduce the precision of prevalence estimates. Fifth, the measurement of bullying in PISA uses behavior-specific indicators rather than formal indicators that feature power imbalance and repetition. Consequently, the identified profiles may represent a broader range of peer victimization (not just narrowly defined bullying). This is relevant when comparing findings and contrasting results from studies based on different measurement frameworks, such as HBSC. This highlights further considerations in cross-national data interpretation.

Finally, the current analyses considered only victimization indicators to identify latent typologies, without including other covariates or distal outcomes. Although this approach is appropriate for setting up a stable and interpretable measurement model, it restricts the analysis of the wider correlates and outcomes of class membership. Past work indicates that bullying profiles are differentially associated with academic achievement, psychological well-being, and school engagement [1,2], indicating that the identified subgroups may also differ in their associated functional outcomes.

Future research will need to build upon the current framework, incorporating auxiliary variables via traditional mixture modeling approaches, such as extending three-step methods (e.g., BCH or R3STEP), that permit investigation of predictors and outcomes while maintaining the integrity of the latent class solution [26]. At the level of large-scale assessment data like PISA, this would allow researchers to model how these victimization profiles are associated with academic performance, well-being outcomes, and school-level climate characteristics, advancing our understanding of educational relevance and informing the focus of interventions.

Future research should extend this work longitudinally to examine transitions among bullying profiles over time and to identify predictors of movement into or out of high-risk subgroups. Incorporating multilevel frameworks that model school-level climate and leadership characteristics may clarify contextual drivers of gender differences. Additionally, integrating academic achievement or well-being outcomes could evaluate the functional consequences of class membership and guide targeted intervention design. Finally, replication in other cultural contexts would determine the generalizability of the observed three-class structure.

In summary, bullying victimization among Saudi students is best understood as a heterogeneous phenomenon composed of discrete subgroups rather than a single continuum. Although males and females exhibit the same latent profiles, they differ in the proportion of students exposed to higher levels of risk. These findings support the use of person-centered approaches for identifying vulnerable students and suggest that educational policies should combine universal prevention with targeted supports.

## Figures and Tables

**Table 1:** Weighted prevalence of specific bullying victimization experiences by gender in Saudi Arabia.

Bullying Behavior	Any Exposure Overall %	Girls %	Boys %	Boys−Girls (pp)	Monthly or More Overall %	Girls %	Boys %	Pearson χ^2^ *p*
Other students made fun of me	24.3	20.4	28.8	8.4	8.2	6.2	10.5	<0.001
Other students spread nasty rumors about me	16.9	16.5	17.3	0.8	5.8	5.3	6.3	<0.001
Other students left me out of things on purpose	13.1	13	13.3	0.2	4.1	3.7	4.5	<0.001
I stayed home from school because I felt unsafe	11.3	11.2	11.5	0.3	5.4	5.1	5.7	<0.001
I was in a physical fight on school property	9.9	4.4	16.2	11.7	3.2	1.2	5.5	<0.001
Other students took away or destroyed things that belonged to me	9.4	6.6	12.7	6.2	3.6	2	5.4	<0.001
I was threatened by other students	9.3	5.3	13.8	8.5	3.3	1.7	5.2	<0.001
I got hit or pushed around by other students	8.2	4.5	12.3	7.8	3.1	1.3	5.2	<0.001
I gave money to someone at school because they threatened me	7.5	3.4	12.1	8.8	5.3	2.4	8.4	<0.001

**Note.** Percentages are based on the final trimmed nonresponse-adjusted student weight. “Any exposure” was defined as endorsement of “A few times a year,” “A few times a month,” or “Once a week or more.” “Monthly or more” was defined as endorsement of “A few times a month” or “Once a week or more.” Pearson chi-square tests were based on the weighted crosstab analyses.

**Table 2:** Model fit using 1–8 classes for females in Saudi Arabia.

Model	LL	npar	AIC	BIC	aBIC	MAIC	AWE	BF (K, K + 1)	cmP(K)	AICc	HQ	Entropy
1	−11,606	27	23,265.93	23,432.20	23,346.40	23,459.20	23,733.46	0.000	0.000	23,266.37	23,325.28	
2	−10,067.4	55	20,244.77	20,583.45	20,408.69	20,638.45	21,197.14	0.000	0.000	20,246.56	20,365.66	0.871
3	−9780.26	83	19,726.52	20,237.63	19,973.90	20,320.63	21,163.74	0.000	0.000	19,730.61	19,908.95	0.846
4	−9633.48	111	19,488.96	20,172.49	19,819.79	20,283.49	21,411.03	>15	1.000	19,496.32	19,732.94	0.857
5	−9551.25	139	19,380.50	20,236.45	19,794.78	20,375.45	21,787.41	>15	0.000	19,392.11	19,686.02	0.865
6	−9500.18	167	19,334.36	20,362.74	19,832.10	20,529.74	22,226.12	>15	0.000	19,351.25	19,701.43	0.87
7	−9470.71	195	19,331.42	20,532.22	19,912.61	20,727.22	22,708.02	>15	0.000	19,354.62	19,760.03	0.867
8	−9433.62	223	19,313.25	20,686.47	19,977.89	20,909.47	23,174.69	0.000	0.000	19,343.83	19,803.40	0.852

**Table 3:** Model fit using 1–8 classes for males in Saudi Arabia.

Model	LL	npar	AIC	BIC	aBIC	MAIC	AWE	BF (K, K + 1)	cmP(K)	AICc	HQ	Entropy
1	−15,273.7	27	30,601.38	30,765.45	30,679.66	30,792.45	31,064.51	0.000	0.000	30,601.86	30,660.19	
2	−11,814.6	55	23,739.16	24,073.37	23,898.61	24,128.37	24,682.57	0.000	0.000	23,741.11	23,858.94	0.934
3	−11,362.3	83	22,890.61	23,394.96	23,131.23	23,477.96	24,314.31	0.000	0.000	22,895.06	23,071.38	0.86
4	−11,081.9	111	22,385.85	23,060.35	22,707.65	23,171.35	24,289.84	0.000	0.000	22,393.86	22,627.60	0.873
5	−10,896.7	139	22,071.33	22,915.96	22,474.30	23,054.96	24,455.60	>15	1.000	22,083.97	22,374.06	0.891
6	−10,820.4	167	21,974.74	22,989.51	22,458.88	23,156.51	24,839.29	>15	0.000	21,993.13	22,338.45	0.898
7	−10,754.8	195	21,899.58	23,084.50	22,464.90	23,279.50	25,244.42	>15	0.000	21,924.87	22,324.27	0.885
8	−10,689.7	223	21,825.44	23,180.51	22,471.94	23,403.51	25,650.57	0.000	0.000	21,858.81	22,311.12	0.876

## Data Availability

Data are available at: https://www.oecd.org/en/data/datasets/pisa-2022-database.html (accessed on 21 April 2026).
